# Is exposure to secondhand smoke associated with current depression (PHQ-8) among never-smokers? Results from a survey among German adults

**DOI:** 10.1186/s12889-020-09970-1

**Published:** 2020-12-07

**Authors:** Fabian Erdsiek, Patrick Brzoska

**Affiliations:** Universität Witten/Herdecke, Alfred-Herrhausen-Strasse 50, 58448 Witten, Germany

**Keywords:** Second-hand smoke, Depression, PHQ-8, Cross-sectional data

## Abstract

**Background:**

Findings on the association between exposure to secondhand smoke (SHS) and depression are contradictory. Comparability of existing research is limited due to varied methods and measures. This study examines the potential association between exposure to SHS and depression and a potential moderation by sex using representative data from Germany.

**Methods:**

For our study, we used data from the German Health Update (GEDA) 2014/2015 on *n* = 10,274 never-smokers. We calculated a logistic regression model with an interaction term for potential sex-exposure interactions. We used the self-reported duration of exposure to predict current depression of any type as defined by the Patient Health Questionnaire PHQ-8 (score ≥ 10), accounting for a large number of confounders.

**Results:**

In our sample, prevalence of depression was 8.8% in women and 5.3% in men. 90.4% were never or almost never exposed to SHS, while 7.1% were exposed < 1 h per day and around 2.5% reported being exposed for ≥1 h per day. While SHS exposure for < 1 h per day was not associated with current depression (OR = 1.54; 95%-CI: 0.93–1.61), SHS exposure for at least 1 h per day was associated with increased odds for current depression (OR = 1.59; 95%-CI: 1.08–2.35). No sex-specific differences were found.

**Conclusions:**

Higher levels of SHS exposure are associated with current depression, although the nature and direction of the association are still unclear. We identified no differences in the association between men and women. More studies, particularly using longitudinal data, are needed to determine the nature of the association.

## Background

Several studies have addressed potential associations between smoking or exposure to secondhand smoke (SHS) and the risk of depressive disorders or depressive symptoms and anxiety [1–3]. While some studies found positive associations between SHS exposure and depression and in some cases identified dose-response relationships [4–6], other studies did not identify such associations [7]. In addition, some studies identified some sex-specific effects or suggested exposure-by-sex interactions [5, 8–11], while other studies did not find any sex-specific differences in the effects [6, 7].

The direction of the association and potential underlying mechanisms are largely unclear [12]. Some explanations assume that SHS exposure reduces the levels of certain neurotransmitters, such as dopamine, serotonine or γ-aminobutyric acid (GABA) [4, 5, 10, 13]. Other hypotheses suggest that involuntary exposure in social situations can cause stress and lead to depressive symptoms [4, 10, 13]. The association between SHS exposure and non-communicable diseases has also been mentioned as a potential explanation, since non-communicable diseases have been linked to depressive disorders as well [6].

The aforementioned studies vary greatly in study design, sample population and measurements of exposure and outcome. While some studies rely on measured information about exposure to smoke [5–7], using different measurements of cotinine, other studies use self-reported exposure as their measurement [9–11]. Similarly, definitions of a positive outcome vary greatly. Some studies use self-reported diagnoses of depression or self-reported depression [4, 9–11], other studies draw on validated psychometric instruments, like the General Health Questionnaire or the Composite International Diagnostic Interview [5–7].

In a recent study using pooled data from Germany, no association was identified when using self-reported diagnosis of depression and self-reported exposure as measurements [14]. Self-reported diagnosis of depression, however, bears the risk of underestimating the prevalence of depression due to social desirability bias and undiagnosed cases of depression. Using representative recent data from Germany, the aim of the present study is to identify a potential association between exposure to SHS and current depression, as defined by a self-assessment by means of the Patient Health Questionnaire 8 (PHQ-8). In addition, we investigated potential sex-specific differences in this association.

## Methods

### Study design

For our study we used secondary data from the fourth wave of the German Health Update (GEDA) survey from 2014/2015 [15, 16]. GEDA is a recurring survey that is conducted by the Robert Koch Institute, the main scientific public health institution of the German Ministry of Health. The GEDA survey is part of regular German health reporting and is designed to be representative of the general population of Germany. It covers a wide range of topics, including health service use, health behavior, sociodemographic and economic aspects and health outcomes.

The GEDA 2014/2015 wave also integrated the European Health Interview Survey (EHIS). The target population for the EHIS is the German-speaking population with a registered private home in Germany aged 15 years or older. For the GEDA dataset, only respondents aged 18 years or older were included. The sample was drawn using a two-stage stratified cluster sampling approach using address registers. The study applied a mixed-mode design, where respondents could participate using either a standardized online questionnaire or a standardized paper-and-pencil questionnaire, according to their preference. The overall sample comprised 24,016 male and female respondents aged 18 years or older, at a response rate of 26.9%. For our analysis we only included never-smokers (*n* = 11,178) and excluded those with missing information on the variables and covariates (*n* = 904), resulting in a subsample of *n* = 10,274.

### Measures and variables

In our data, overall exposure to secondhand smoke was measured using self-reported duration of exposure to secondhand smoke in closed rooms per day. Categories of the variable were “never or almost never”, “less than 1 hour per day” and “1 hour per day or more”. To measure the outcome of depression, we used the Patient Health Questionnaire 8 (PHQ-8), which is widely used to screen for depressive symptoms and indications of depressive disorders in different countries and cultural settings [17–19]. The PHQ-8 is a short version of the PHQ-9, omitting one item asking about suicidal ideation, and has generally similar diagnostic results [20–22]. The PHQ-8 distinguishes between respondents who currently have no indication of depression, indication of major depression and indication of other types of depression [22, 23]. Its total score ranges between 0 and 24. In line with recommendations, we used a cut-off value of ≥10 points to determine indication of current depression [23]. To adjust for potential confounding we included information on *sex* (male/female), *age* (in 5-year age groups), *socioeconomic status* (low/medium/high) -- a standardized summary measure based on occupational status, vocational education and net equivalent income [24] –, *place of residence* (Eastern/Western Germany); *degree of urbanization of the place of residence* (district-free city/urban district/rural district with densely populated areas/sparsely populated rural district), *partnership status* (with a partner/without a partner), *social support* according to the Oslo Social Support Scale (OSS-3) (low/moderate/high support) [25], *alcohol consumption* (no regular consumption/no weekly risky consumption/risky consumption), *body mass index* (< 25/ > = 25) and the *presence of chronic diseases* (yes/no).

### Statistical analysis

Descriptive statistics (uni- and bivariate) were used to describe the structure of the sample population. To identify bivariate differences, χ^2^-tests were used. We applied multivariable binary logistic regression modelling to identify a potential association between exposure to secondhand smoke and depression while adjusting for confounders. Associations are presented as adjusted Odds Ratios (OR) with 95%-confidence intervals. As a sensitivity analysis we performed a multiple linear regression with robust standard errors using the log-transformed PHQ-8 sum score. To identify possible sex-by-exposure interactions, i.e. differences in effect sizes between men and women, we included interaction terms into the model and calculated predicted margins and marginal contrasts [26, 27]. All analyses were done using Stata 15 [28].

## Results

Our sample consisted of 3956 men and 6318 women. Overall prevalence of any type of depression in our sample as determined by a PHQ-8 score of ≥10 was 7.4%, with a higher prevalence in women than in men (8.8% vs. 5.3%). While the majority were never or almost never exposed to SHS (90.4%), 7.1% were exposed less than 1 h per day and around 2.5% reported being exposed for 1 h per day or longer. Overall, men had higher percentages of exposure. While 9.6% of men reported medium exposure and 2.7% high exposure, only 5.6% of women reported medium exposure and 2.4% high exposure.

With increasing exposure to SHS the prevalence of depression among participants increased, from 7.0% among those never or almost never exposed to 10.2% among those exposed less than 1 h per day and 14.1% among those exposed for 1 h per day or longer (*p* < 0.001). We also found significant differences between exposure groups for all covariates, with the exception of the degree of urbanization (*p* = 0.292) (Table 1).

**Table 1:** Sample characteristics by duration of exposure to secondhand smoke (SHS) (data from the 2014/2015 German Health Update [GEDA] survey on all never-smokers; *n* = 10,274)

	Exposure to SHS								***p***-value
	never or almost never (*n* = 9286)		less than 1 h per day (*n* = 732)		1 h per day or more (*n* = 256)		total (*n* = 10,274)		
	n	%	n	%	n	%	n	%	
**Sex**									
female	5816	62.6%	351	48.0%	151	59.0%	6318	61.5%	< 0.001*
male	3470	37.4%	381	52.0%	105	41.0%	3956	38.5%	
**Socioeconomic status**									
low	1136	12.2%	138	18.9%	75	29.3%	1349	13.1%	< 0.001*
medium	5046	54.3%	459	62.7%	153	59.8%	5658	55.1%	
high	3104	33.4%	135	18.4%	28	10.9%	3267	31.8%	
**Partnership status**									
with a partner	5439	58.6%	236	32.2%	112	43.8%	5787	56.3%	< 0.001*
without a partner	3847	41.4%	496	67.8%	144	56.3%	4487	43.7%	
**Place of residence**									
Western Germany	7006	75.4%	515	70.4%	191	74.6%	7712	75.1%	0.009*
Eastern Germany	2280	24.6%	217	29.6%	65	25.4%	2562	24.9%	
**Degree of urbanization**									
district-free city	2753	29.6%	215	29.4%	78	30.5%	3046	29.6%	0.292
urban district	3441	37.1%	272	37.2%	100	39.1%	3813	37.1%	
rural district with densely populated areas	1475	15.9%	110	15.0%	26	10.2%	1611	15.7%	
sparsely populated rural district	1617	17.4%	135	18.4%	52	20.3%	1804	17.6%	
**Social support (OSS 3)**									
low	1411	15.2%	151	20.6%	42	16.4%	1604	15.6%	0.006*
moderate	5097	54.9%	402	54.9%	156	60.9%	5655	55.0%	
high	2778	29.9%	179	24.5%	58	22.7%	3015	29.3%	
**Alcohol consumption**									
no regular consumption	5311	57.2%	409	55.9%	163	63.7%	5883	57.3%	< 0.001*
no weekly risky consumption	2984	32.1%	226	30.9%	53	20.7%	3263	31.8%	
risky consumption	991	10.7%	97	13.3%	40	15.6%	1128	11.0%	
**Body mass index**									
< 25	4285	46.1%	331	45.2%	159	62.1%	4775	46.5%	< 0.001*
≥ 25	5001	53.9%	401	54.8%	97	37.9%	5499	53.5%	
**Presence of chronic diseases**									
yes	3932	42.3%	269	36.7%	117	45.7%	4318	42.0%	< 0.001*
no	5354	57.7%	463	63.3%	139	54.3%	5956	58.0%	
**Current depression (PHQ-8 ≥ 10)**									
**yes**	**652**	**7.0%**	**75**	**10.2%**	**36**	**14.1%**	**763**	**7.4%**	< 0.001*
**no**	**8634**	**93.0%**	**657**	**89.8%**	**220**	**85.9%**	**9511**	**92.6%**	

*OSS 3* Oslo Social Support Scale 3, *PHQ* Patient Health Questionnaire

* significant differences between exposure groups at 5%-level

After adjusting for potential confounders, logistic regression modeling of the main effects showed no significant association between exposure to SHS for less than 1 h per day and indication of depression (OR = 1.22; *p* = 0.154) (Table 2). In contrast, exposure to SHS for 1 h per day or longer was significantly associated with an increased odds of currently suffering from depression (OR = 1.59; *p* = 0.018). As a sensitivity analysis we calculated a multiple linear regression model with robust standard errors and using log-transformed PHQ-8 sum scores as the dependent variable to meet all necessary assumptions. The sensitivity analysis showed significantly higher scores for exposure to SHS for less than 1 h per day (β = 0.1; *p* = 0.001) and exposure to SHS for 1 h per day or longer (β = 0.15; *p* = 0.001).

**Table 2:** Results of the multivariable logistic regression model with current depression (PHQ-8 ≥ 10) as the dependent variable (data from the 2014/2015 German Health Update [GEDA] survey on all never-smokers; *n* = 10,274; main effects model, no interaction effects included)

		Odds Ratio	*p*-value	95% confidence interval	
**Exposure to SHS**	**Never or almost never (ref.)**	***1.00***			
	**Less than 1 h per day**	**1.22**	**0.154**	**0.93**	**1.61**
	**1 h per day or more**	**1.59**^**8**^	**0.018**	**1.08**	**2.35**
Sex	Male (ref.)	*1.00*			
	Female	1.61*	< 0.001	1.35	1.92
Age	(in 5-year groups)	0.95*	< 0.001	0.92	0.97
Partnership status	With a partner (ref.)	*1.00*			
	Without a partner	1.49*	< 0.001	1.26	1.76
Socioeconomic status	Low (ref.)	*1.00*			
	Medium	0.72	0.001	0.58	0.88
	High	0.52*	< 0.001	0.41	0.67
Social support (OSS 3)	Low support (ref.)	*1.00*			
	Moderate support	0.34*	< 0.001	0.28	0.40
	High support	0.20*	< 0.001	0.16	0.25
Place of residence	Western Germany (ref.)	*1.00*			
	Eastern Germany	0.91*	0.327	0.75	1.10
Degree of urbanization	District-free city (ref.)	*1.00*			
	Urban district	0.81	0.039	0.67	0.99
	Rural district with densely populated area	0.90	0.381	0.71	1.14
	Sparsely populated rural district	0.90	0.398	0.71	1.14
Presence of chronic diseases	Yes (ref.)	*1.00*			
	No	0.28*	< 0.001	0.24	0.34
Body mass index	25 or higher (ref.)	*1.00*			
	Less than 25	0.93	0.425	0.79	1.10
Alcohol consumption	No regular consumption (ref.)	*1.00*			
	No weekly risky consumption	0.63*	< 0.001	0.52	0.77
	Risky consumption	0.80	0.120	0.61	1.06

*SHS* Secondhand smoke, *OSS 3* Oslo Social Support Scale 3, *PHQ* Patient Health Questionnaire, *ref.* reference category

* statistically significant at 5%-level

The logistic regression model further revealed that being female was associated with increased odds of depression as well (OR = 1.61; *p* < 0.001). We also found significant associations for the majority of covariates, apart from those relating to place of residence, weight and risky alcohol consumption. To determine potential sex-specific differences in the association, we fitted a model using interaction terms and calculated predictive margins and marginal contrasts, but did not identify any significant interaction in the association (not shown).

## Discussion

The main aim of our study was to examine the association between exposure to secondhand smoke (SHS) and current depression. Using data from a recent, nationally representative German survey and focusing on never-smokers, we observed a positive association between higher levels of exposure to SHS and current depression as measured by the PHQ-8. While SHS exposure of < 1 h/day, as compared to no exposure, was associated with 22% higher odds of depression (however, not statistically significant), never-smokers exposed ≥1 h/day per day had 59% significantly higher odds of depression than never-smokers not exposed to SHS. A sensitivity analysis also showed a significant positive correlation between levels of exposure to SHS and PHQ-8 scores. This corresponds to findings of positive associations for different populations from other studies [5, 6]. Another recent study among never-smokers also identified positive associations between exposure to SHS and depressive symptoms and established dose-response relations [4]. We found no differences in the association between men and women. This is in line with some of the findings from other studies [1, 6], but not with others [5, 9–11].

As mentioned before, different hypotheses have been presented to explain the association between exposure to SHS and depression, with some of these not being mutually exclusive. Under the assumption of a causal effect of exposure to SHS on the risk of depression and/or symptom severity, public health policies to reduce passive smoking might, among other health benefits, contribute to reducing the burden of depression in the German population. One study from Germany found that the main area of exposure to SHS was the workplace, suggesting the necessity for further restrictions to smoking in public [29]. Other frequently reported places of exposure were bars/discotheques and the home of a friend, suggesting that non-smokers do not actively avoid exposure in these situations [29]. In light of these findings, psychotherapeutic approaches to depression could be supplemented by addressing voluntary social interactions that include passive smoking. For active smoking, some studies suggest bidirectional effects [30–33], where smoking is used to self-medicate but also affecting levels of neurotransmitters and so creating an additional motive to increase or continue tobacco consumption. Other studies have suggested that bidirectional associations may result at least partially from shared underlying risk factors, such as genetic factors and gene-environment interactions [14, 34, 35].

Previous research has been diverse in terms of measurements used and populations investigated. This limits the comparability across studies. In a previous study that used an earlier wave of the GEDA survey, no association was found when using self-reported medical diagnosis of depression as the outcome [14]. This could suggest an underestimation due to undiagnosed cases of depression. In addition, individuals with a diagnosis of depression are likely to get medical and/or psychotherapeutic treatment, which could affect the association due to lower symptom levels among diagnosed as compared to undiagnosed individuals. Similar to other studies [4–6], we identified a positive association between higher levels of SHS exposure and depression using the PHQ-8. In this respect, measurements of depression that detect lower levels of symptoms might be better suited to identify associations between exposure to SHS and depression.

This study has several limitations that need to be considered. Due to the use of cross-sectional data from a survey, we cannot establish any causality or timeline. We used self-reported duration on SHS which may have over- or underestimated the true exposure. Social desirability bias may lead to active smokers being classified as non- or never-smokers and overestimation of the association. The strengths of our study include the use of a large, population-based dataset that is representative for German adults. Another strength is the use of a validated psychometric instrument to assess depression instead of self-reported diagnosis of depression or non-validated instruments. We also included a large number of known confounders and considered sex-specific differences in the association.

## Conclusions

We found positive associations between higher levels of self-reported exposure to second-hand smoke and current depression as determined by the PHQ-8. We identified no significant differences between men and women in the strength of the association. More studies, preferably using longitudinal data and validated measures of depression or depressive symptoms, are necessary to identify the directionality or bidirectionality of the association and potential causal effects. The association of exposure to SHS and depression suggests that measures to reduce exposure to SHS may contribute to reducing the burden of mental illness in addition to other health benefits.

## Data Availability

The dataset analyzed in the current study is available from the Robert Koch-Institute upon application: https://www.rki.de/EN/Content/Health_Monitoring/Public_Use_Files/application/application_node.html.

## Abbreviations

EHIS: European Health Interview Survey
GABA: γ-aminobutyric acid
GEDA: German Health Update
OR: Odds Ratio
OSS-3: 3-item Oslo Social Support Scale
PHQ-8: 8-item version of the Patient Health Questionnaire
SHS: Second-hand smoke
